# Defining correlates of protection for mammalian livestock vaccines against high-priority viral diseases

**DOI:** 10.3389/fimmu.2024.1397780

**Published:** 2024-07-19

**Authors:** Samantha K. Davis, Fan Jia, Quentin G. Wright, Md. Tanjir Islam, Andrew Bean, Daniel Layton, David T. Williams, Stacey E. Lynch

**Affiliations:** Commonwealth Scientific and Industrial Research Organisation (CSIRO) Australian Centre for Disease Preparedness, Geelong, VIC, Australia

**Keywords:** correlates of protection, livestock, biosecurity, vaccines, protection, immunity, antibodies, T cells

## Abstract

Enhancing livestock biosecurity is critical to safeguard the livelihoods of farmers, global and local economies, and food security. Vaccination is fundamental to the control and prevention of exotic and endemic high-priority infectious livestock diseases. Successful implementation of vaccination in a biosecurity plan is underpinned by a strong understanding of correlates of protection—those elements of the immune response that can reliably predict the level of protection from viral challenge. While correlates of protection have been successfully characterized for many human viral vaccines, for many high-priority livestock viral diseases, including African swine fever and foot and mouth disease, they remain largely uncharacterized. Current literature provides insights into potential correlates of protection that should be assessed during vaccine development for these high-priority mammalian livestock viral diseases. Establishment of correlates of protection for biosecurity purposes enables immune surveillance, rationale for vaccine development, and successful implementation of livestock vaccines as part of a biosecurity strategy.

## 1 Introduction

**Figure 1:**
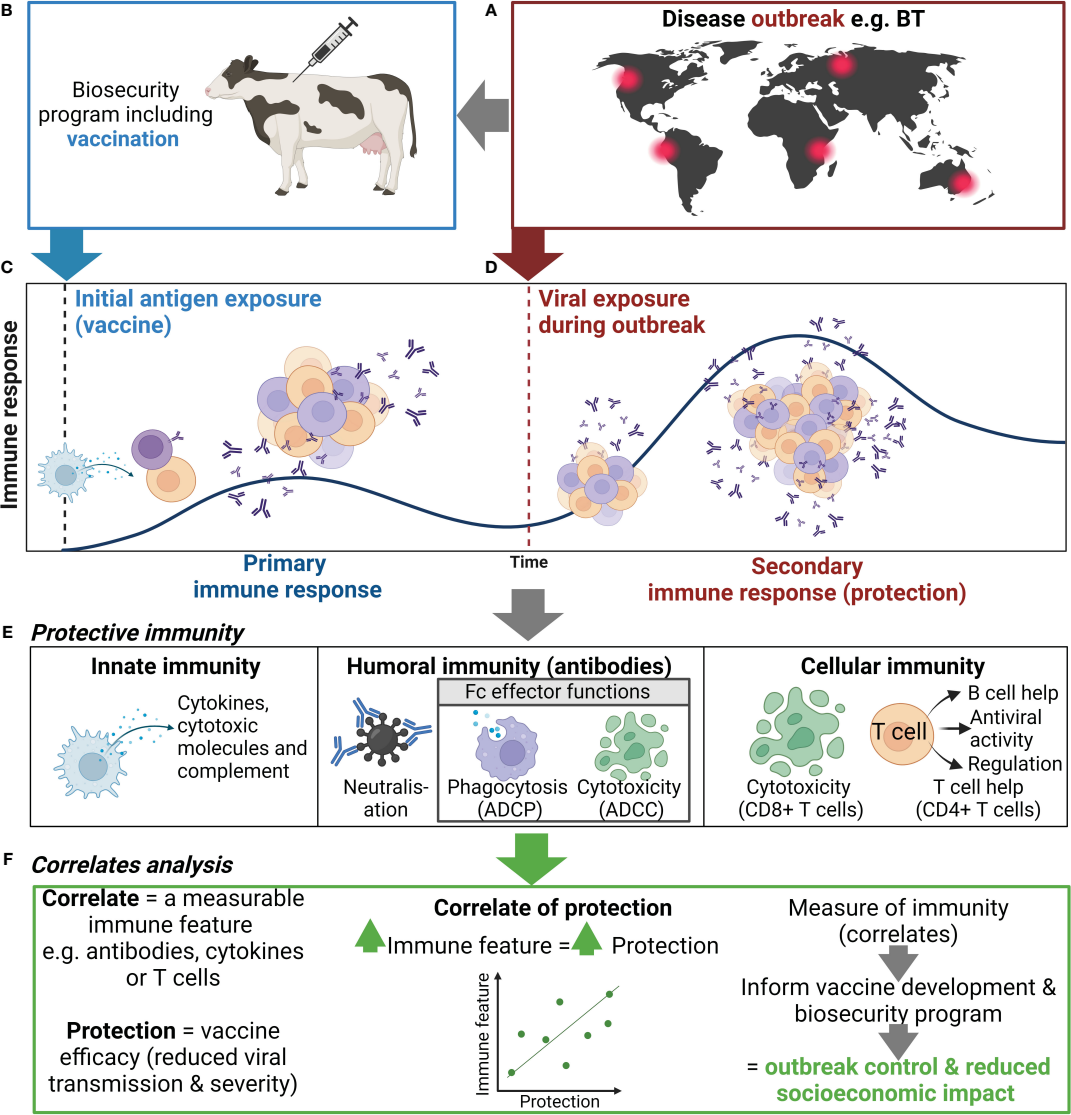
Use of correlates of protection to inform biosecurity programs. **(A)** An outbreak of an important viral livestock disease, e.g., bluetongue (BT), **(B)** triggers the implementation of a biosecurity program that may include vaccination. **(C)** Initial exposure to antigen via vaccination or natural infection will generate a primary immune response to the target virus, including activation, proliferation, and differentiation of innate (e.g., NK cells and DCs) and adaptive immune cells (e.g., B and T cells), and the production of antibody and generation of immune memory. **(D)** When immune animals encounter a subsequent infection, immune memory rapidly activates and expands to generate an efficient secondary immune response. **(E)** The antiviral immune response elicits various functions including cytokine secretion, antibody-mediated neutralization, and antibody Fc effector functions {i.e., antibody-dependent cellular phagocytosis (ADCP), cytotoxicity of infected cells [antibody-dependent cellular cytotoxicity (ADCC)], and CD8+ T cell mediated}, which collectively controls viral replication and clears infection, thus reducing disease severity and ongoing viral transmission. **(F)** Correlating these immune features can help identify indicators of protection to inform vaccine development and the use of vaccination in biosecurity programs. Created with BioRender.com.

Biosecurity involves implementation of measures to minimize the risk of introducing and spreading pathogens within and between farms, between humans and animals, and within the environment (1, 2). The development of biosecurity programs requires a strong scientific basis and the use of risk assessment to evaluate and implement appropriate measures without unnecessarily hindering commerce and trade (1, 2). Many measures are utilized to prevent and control exotic and endemic high-priority viral animal diseases ranging from animal husbandry practices to culling of infected animals or herds (1, 2). Livestock vaccines can further bolster biosecurity strategies and contribute significantly to the overall health and productivity of animal populations (**Figure 1**) (3, 4). While the immune response to infection with many high-priority viral livestock diseases is well characterized, the protective immune response to a subsequent infection and the relationship to vaccine-mediated protection are incomplete. In this review, we discuss current knowledge of protective immunity and vaccine-induced correlates of protection (CoPs) for high-priority mammalian livestock viral diseases in the context of biosecurity (**Table 1**). We selected six examples of viral diseases notifiable to the World Organization for Animal Health (WOAH), namely, foot and mouth disease (FMD), African swine fever (ASF), lumpy skin disease (LSD), bluetongue (BT), porcine reproductive and respiratory syndrome (PRRS), and classical swine fever (CSF) (**Table 1**). These specific diseases were selected as they are high-consequence viral diseases in mammalian livestock, for which vaccination is or aims to be employed as part of a biosecurity and control strategy. Furthermore, these diseases represent diversity in the presentation of disease and the immune responses associated with protection and include both RNA and DNA viruses.

**Table 1:** Summary table of evidence for innate, humoral, and cellular immune features as correlates of protection against viruses causing disease of livestock.

Virus	Host(s)	Type of vaccine	Vaccine efficacy	Strength of evidence as correlate of protection from licensed vaccines and vaccines in development				
				Innate		Humoral		Cellular
				Cytokines	NK cells	Neutralizing antibodies	Fc effector functions	T cells
**LSDV**	Bovine species	Live-attenuated LSDV*	Mod (5, 6).	Potential (7–9)	Unknown	Limited (IgM) (10, 11)	Unknown	Potential (9, 11)
**BTV**	Wild and domestic ruminants	Inactivated virus*	High ^A^ (12)	Potential (13, 14)	Unknown	Established (15, 16)	Unknown	Potential (14)
		Live-attenuated BTV*	High (12, 17)	Potential (18–20)	Unknown	Potential (20–22)	Unknown	Unknown
		Viral-vectored	Low–Mod (23–25).	Potential (23)	Unknown	Potential (25, 26)	Unknown	Potential (23, 25)
		DNA	Mod (23).	Potential (23)	Unknown	Unknown	Unknown	Potential (23)
		Passive immunization	Mod.–High (27, 28)	NA	NA	Established (27, 28)	Unknown	Potential (29)
		Infection in resistant animals	Low–Mod (30, 31).	Potential (30)	Unknown	Potential (31)	Unknown	Potential (31)
**PRRSV**	Swine	Inactivated virus*	Mod (32–34).	Potential (35–38)	Potential (35)	Established (32, 34, 38–41)	Unknown	Potential (42)
		Live-attenuated*	Mod (32, 43).	Potential (35–37, 44)	Potential (35)	Potential (32, 45, 46)	Unknown	Potential (44, 45, 47–50)
		Recombinant protein*	Low–Mod (51–53).	Unknown	Unknown	Established (51)	Unknown	Potential (52, 53)
		Passive immunization	High (54, 55)	NA	NA	Established (54, 55)	Unknown	Unknown
**FMDV**	Biungulate species	Inactivated virus*	Low ^c^ (56, 57)	Established (IFNγ); Potential (58–61)	Unknown	Established (62–64)	Potential (65)	Potential (58, 61, 66–68)
		Viral-vectored	High (56, 69, 70)	Established (IFNγ); Potential (71–73)	Unknown	Potential (74–77)	Unknown	Potential (74, 78, 79)
		DNA	High (80)	Potential (80)	Unknown	Potential (80)	Unknown	Potential (80)
		Passive immunization	Mod (81–83).	NA	NA	Established (81)	Potential (82, 83)	Unknown
**ASFV**	Wild and domestic pig species	Live-attenuated ASFV*	Mod.–High (84–86)	Potential (87–89)	Potential (89, 90)	Limited (88, 91)	Unknown	Potential (87–89, 92–94)
		Recombinant protein	Low (95)	Unknown	Unknown	Potential (95)	Unknown	
		DNA	Low (96)	Unknown	Unknown	Limited potential (96)	Unknown	Potential (96)
		Viral-vectored	Low-High	Potential (IFNγ) (97, 98)	Unknown	Limited potential (97, 99, 100)	Potential (99)	Potential (97, 98)
		Inactivated virus	Low/no (101–103)	NA	NA	NA	NA	NA
		Passive immunization	Mod (104–106).	NA	NA	Limited (106)	Potential (104, 105)	Unknown
**CSFV**	Wild and domestic pig species	Live-attenuated CSFV*	High (107, 108)	Established (IFNγ); Potential (109)	Unknown	Established (110, 111)	Unknown	Potential (112–114)
		Recombinant protein E2*	High (107, 115)	Established (IFNγ); Potential (114, 116–118)	Unknown	Established (117)	Unknown	Potential (117)
		DNA (E2)	High (113)	Unknown	Unknown	Potential (113)	Unknown	Potential (113)
		Nanoparticle (E2)	High (119)	Potential (119)	Unknown	Potential (119)	Unknown	Potential (119)
		Passive immunization	High (120)	NA	NA	Established (120)	Unknown	Unknown

High: High level of protection defined as sterilizing or near-sterilizing immunity with high level of protection from clinical signs and absence or reduced viremia.

Mod.: Medium level of protection defined as high level of protection from clinical signs but minimal control of viremia.

Low: Low level of protection defined as protection from clinical signs but no–minimal control of viremia with vaccinated animals can act as carriers, and potential for viral shedding.

Established: Immune factor has been assessed and determined as a correlate of protection on several occasions.

Limited: Immune factor has been assessed as a correlate; however, results are inconclusive or variable.

Potential: Immune factor is elevated in protected animals compared to non-protected; however, it is yet to be assessed as a correlate of protection.

Limited Potential: Immune factor is sometimes elevated in protected animals compared to non-protected; however, results are inconclusive or variable and yet to be assessed as a correlate of protection.

Not applicable (NA): The immune factor of interest can not be assessed against protection either due to lack of protection or a lack of the immune factor in passive immunization.

Unknown: Immune factor is yet to be comprehensively studied in the context of protection and immune correlate analysis has not been complete.

*Licensed vaccine.

## 2 Vaccination in response to high-priority livestock viral diseases

Viral vaccines typically comprise a part of the virus (e.g., sub-unit or recombinant protein), whole viral particles with reduced virulence [e.g., live-attenuated viral (LAV) vaccine], or inactivated viral particles (inactivated vaccine) (121). For the example diseases discussed here, licensed vaccines are most commonly LAVs or inactivated vaccines; however, subunit vaccines for PRRSV and CSFV have also been licensed (**Table 1**). LAVs generally induce strong cellular and humoral immune responses compared to inactivated and subunit vaccines (13, 122, 123). Inactivated and subunit vaccines generally confer protection through humoral immunity; however, inclusion of adjuvants, appropriate antigen selection, and additional doses can induce cell-mediated immunity (CMI) (13, 122, 123) (**Table 1**). Vaccination can be applied as part of a response to an incursion of a high-priority viral disease to limit the spread and eventually eradicate the disease (**Figures 1A, B**) (124, 125). For example, cattle plague caused by rinderpest virus led to an approximately 20% loss of dairy cattle in Europe during the 20th century (126). The elimination of rinderpest virus was achieved in 2011 through multi-organizational campaigns implementing vaccines as a prevention measure as part of a global biosecurity plan (126). Furthermore, control of FMD outbreaks has been achieved through vaccination programs in South Africa and Uruguay (127, 128). Livestock vaccination is an effective biosecurity measure to reduce the economic impact of high-priority livestock diseases (**Figure 1**). However, the implementation of such vaccines varies between countries and is contingent on various factors including cost, vaccine availability, epidemiological factors, regulatory considerations, and host distribution, and may require vaccine matching to the outbreak strain. For example, a LAV ASFV vaccine has recently been approved for use in Vietnam to help control this devastating virus (129). However, in ASF-free countries such as Australia, this vaccine is not approved due to the risks associated with the use of an ASFV LAV (e.g., reversion to virulence) and is not currently part of Australia’s emergency response plan to an ASF outbreak (130). A major advantage of non-LAV or inactivated vaccine platforms is that these vaccines can be designed in parallel with DIVA diagnostic assays to help inform the culling strategy of an outbreak in an endemic area and facilitate trade.

## 3 Defining correlates of protection

In addition to clinical signs, protection can be defined by duration and levels of viremia, viral load in tissues, shedding, and transmission to naive or unvaccinated bystander animals (131). Notably, induction of sterilizing immunity (i.e., preventing replication of the virus) following vaccination is the gold standard level of protection (132). Measurable immune features or correlates, including antibodies, cytokines, or T cells, which are statistically associated with and can reliably predict protection, are defined as CoPs (**Figures 1E, F**) (131). Many factors influence the contribution of an immune feature to protection, including the vaccine delivery platform, antigen(s), host genetics, and the target viral disease (133). CoP analysis assesses the strength of the relationship between the immune feature and protection using animals with varying levels of protection—either immunized/vaccinated animals or naturally resistant animals (131, 134). Single or multiple immune features may be associated with protection for a given disease or vaccine.

Studying the levels of protection achieved through active and passive (transfer of immune sera or antibodies) immunization (134) can provide key insights into the immune response associated with protection and can enable identification of CoPs (**Table 1**). Notably, much of what is known currently about protective immunity against the focus diseases in this review has come from research on vaccine candidates currently in development, with the exception of commercial vaccines listed in **Table 1**.

## 4 Innate immune features as correlates of protection

### 4.1 Antigen-presenting cell function and counts

Depletion of key antigen-presenting cells including macrophages, monocytes, and/or DCs transiently occurs in animals during infection with ASFV, PRRSV, BTV, FMDV, LSDV, and CSFV (135–138). Depletion of these immune cell subsets during acute infection ultimately hinders the development of protective immunity. Furthermore, downregulation of MHC class I and/or MHC class II expression during FMDV (139), ASFV (140), and PRRSV (141) infection hampers antigen presentation and impairs T-cell responses. Therefore, measuring the number, viability, and MHC expression of these cells could act as an early indicator of vaccine success or failure (**Figures 1C-E**). However, such changes in cell counts can vary with strain and virulence, as seen in ASFV infection of pigs (142).

### 4.2 Natural killer cells

Natural killer (NK) cells elicit cytotoxic functions through secretion of cytotoxic molecules (perforin and granzyme) and cytokines, including interferon gamma (IFNγ) and tissue necrosis factor alpha (TNFα) (143). NK cells mediate cytotoxicity directly via cytotoxic molecules or indirectly via antibodies (see Section 5.2) (**Figure 1E**). NK responses are suppressed following infection with ASFV, FMDV, and PRRSV; however, vaccination can retain NK cell activity (35, 90, 144). Notably, protected pigs showed elevated NK cell cytotoxicity and cytokine secretion compared to non-protected pigs following PRRSV LAV vaccination (**Table 1**) (35). Similarly, enhanced NK cell activity has also been observed in pigs immunized with a low virulent strain of ASFV upon subsequent challenge with a highly virulent strain, compared to unimmunized pigs (**Table 1**) (90). Finally, NK cells have shown high levels of cytotoxicity *in vitro* in bovine and porcine cells infected with FMDV (144, 145). Collectively, the literature suggests that the functional response and frequency of NK cells could be assessed as an indicator of vaccine-mediated protection in the host for ASFV, FMDV, and PRRSV. However, the contribution of NK cells in protective immunity remains to be assessed for CSFV, LSDV, and BTV (**Table 1**).

### 4.3 Type I interferon

Viral proteins frequently inhibit the host’s Type I interferon (IFN) production during natural infection via various mechanisms (146–151). Type I IFNs, including IFNα and IFNβ, are anti-viral cytokines secreted by monocytes, macrophages, DCs, and NK cells. *In vitro* studies where virus-infected cells were treated with Type I IFNs have highlighted the potential for these cytokines to inhibit viral replication of BTV (152), ASFV (153), PRRSV (154), CSFV (155), and FMDV (156). Similarly, elevated Type I IFNs have been implicated in protection from homologous challenge following vaccination for BTV (13, 23) and PRRSV (157). Type I IFN adjuvanted vaccines have protected animals from clinical signs and viremia early post-vaccination in the absence of humoral immunity for FMDV (71, 72) and CSFV (116), providing strong evidence for the contribution of Type I IFN in early protective immunity. Interestingly, the contribution of Type I IFN to protection against FMDV appears to be host specific. Elevated levels of protection have been demonstrated in pigs in contrast to cattle using Adenovirus (Ad5) vectored vaccination adjuvanted with host-specific Type I IFN (71, 72). Notably, elevated levels of Type I IFNs following vaccination—either generated by the vaccine or the adjuvant—can enhance the antibody response, which can subsequently promote a long-lived adaptive immune response (116, 158). The potential role of Type I IFNs in early protection from infection with BTV, PRRSV, CSFV, and FMDV is well established (**Table 1**). As for ASFV, IFNα may play a role in vaccine-induced recall response to challenge (87), although the definitive role of IFN and other cytokines in protection from ASFV requires further investigation in protected animals. Similarly, the role of Type I IFN in protection from LSDV has not been assessed and should be investigated further.

### 4.4 Balancing immunopathology and protective immunity

Clinical signs associated with systemic disease caused by ASFV (159–161), BTV (30, 162, 163), PRRSV (146, 164), LSDV (165), and CSV (166, 167) are commonly associated with a “cytokine storm” where overproduction of pro-inflammatory cytokines occurs, leading to extensive immunopathology. The “cytokine storm” has not been reported for FMDV (168). Infection of natural hosts with virulent strains of these viruses have been associated with high levels of cytokines including IL-6, TNFα, IL-1β, IL-8, IFNγ, and/or Type I IFNs (169–178). However, when pro-inflammatory cytokines are generated in a regulated manner to these viruses, they have anti-viral potential (13, 30, 73, 117, 173, 179, 180) and are essential to orchestrating the adaptive protective immune response (87, 181) (**Figure 1E**) (**Table 1**). Measurement of cytokines, such as interleukin-2 (IL-2) or IFN gamma (IFNγ), are commonly used as a surrogate measure of CMI and vaccine immunogenicity (182–184). Most notably, protection from FMDV and CSFV has been correlated to IFNγ secretion by T cells (58, 73, 109). Similarly, IFNγ secretion by T cells has also been linked to protection from PRRSV, but does not fully predict protection (36). Furthermore, elevated levels of these cytokines post-vaccination are not always a reliable indicator of protection. Several candidate ASFV vaccines are considered immunogenic and induce a robust IFNγ response but are not protective (88, 160, 185). Owing to secretion of IFNγ by multiple immune cell subsets (various T cells, macrophages, NK cells, etc.), caution must be taken when making conclusions about the role of specific subsets in protection based on such data. Importantly, protection elicited by the innate immune response is not strain specific and, thus, may generate cross-protection to viruses with high antigenic diversity, including BTV, ASFV, and FMDV, and should be studied using vaccinated protected natural hosts (14, 87). The role of cytokines in protection from LSDV remains a large gap in knowledge and, thus, should be studied further (7–9). It is important to note that many different cytokines have a role in protective immunity for these diseases, and therefore, research should aim to investigate cytokines beyond IFNγ as CoPs (30, 37, 44, 59, 60, 118).

## 5 Humoral immune features as correlates of protection

Following vaccination against a virus of interest, B cells will become activated and differentiate into long-lived memory B cells or short-lived antibody secreting cells called plasmablasts. Antibodies generated from these cells recognize specific epitopes of viral antigens that are exposed on the virion or expressed on the cell surface of infected cells. Antibodies can occur as neutralizing or non-neutralizing antibodies and can elicit various protective immune functions.

### 5.1 Neutralizing antibodies

The most-utilized CoPs are neutralizing antibody (Nab) titers, with higher antibody titers commonly linked to increased protection (**Figures 1E, F**). Reduced viral loads and the absence of clinical disease have consistently been associated with higher Nab titers following passive immunization or vaccination against FMDV (sheep, cattle, and pigs) (62–64, 81), PRRSV (15, 32, 51, 54, 55), BTV (15, 26, 27, 31), and CSFV (107, 110, 111, 117, 120) (**Table 1**). Sterilizing immunity is associated with high Nab titers for PRRSV (54, 186), CSFV (187), and BTV (188, 189). Notably, the longevity of the vaccine-induced Nab response can vary among different vaccines for different diseases (189, 190). While strong Nab responses to PRRSV, FMDV, and BTV can induce strong protection to homologous strains, vaccine-induced protection mediated by Nabs is often strain/genotype specific, due to high levels of antigenic diversity. However, induction of broadly reactive Nabs following active or passive immunization can confer high levels of cross-protection against different strains of PRRSV and CSFV (55, 120, 191). It is also important to note that vaccination against PRRSV, FMDV, and BTV in several instances can elicit protection in the absence of Nabs (192–197).

The role of Nabs in protection from ASFV is a topic of contention. While protection from lethal ASFV infection has been correlated to Nab titers by Silva et al. (2022) (91) following LAV vaccination, many other studies have reported that Nabs were not sufficient for protection following vaccination, for example, with subunit vaccines (**Table 1**) (198, 199). Similarly, while Nabs can protect cattle against LSDV infection, Nabs are not a reliable indicator of protection following vaccination against LSDV as not all vaccinated animals that are fully protected against LSDV seroconvert (10, 11) (**Table 1**). This highlights the risk of assessing Nab titers as the sole CoP in all contexts.

### 5.2 Non-neutralizing antibodies and Fc effector functions

Antibodies bound to viral particles or viral antigens expressed on infected cells engage with Fc receptors (FcRs) on immune cells (e.g., macrophages, DCs, monocytes, and NK cells), resulting in the activation of Fc effector functions. Fc effector functions include antibody-dependent cellular phagocytosis (ADCP) and antibody-dependent cellular cytotoxicity (ADCC), which clear viral particles and kill infected cells to reduce disease severity (**Figure 1E**) (200, 201). Furthermore, Fc effector functions can enhance cross-protection due to the combined breadth of antibody targets recognized by neutralizing and non-Nabs (202, 203). While the mechanism of protection mediated by non-Nabs is poorly understood, phagocytosis has been shown to play a role. Phagocytosis of FMDV by macrophages was observed in mice that received FMDV non-neutralizing monoclonal antibody treatment and were protected from FMDV challenge, compared to untreated mice that succumbed to infection (65, 82, 83). ADCC has been identified as a potential CoP for ASFV. Elevated levels of ADCC were observed in surviving pigs following passive transfer of anti-ASFV immune sera and following serial immunization with attenuated ASFV compared to non-immunized susceptible pigs (99, 104, 105). However, the contribution of ASFV-specific ADCC to protection is likely vaccine dependent (204).

In rare instances, vaccine-induced antibodies may play a role in antibody-dependent enhancement (ADE) of infections where viral immune complexes are phagocytosed by immune cells and lead to a productive infection (205). ADE in livestock viral infections has been speculated for ASFV, PRRSV, and FMDV (206–208). Most literature surrounding ADE stems from *in vitro* assays where enhanced levels of viral replication have been detected in the presence of serum antibodies (206). Although well described for dengue virus (209), ADE remains controversial for these livestock diseases as the small number of *in vivo* studies reported have shown limited evidence of ADE occurring (206, 210).

Research on Fc effector functions such as CoPs has been limited or absent for ASFV, CSFV, BTV, LSDV, PRRSV, and, to a lesser extent, FMDV (**Table 1**). Thus, there is a need to characterize the role of vaccine-induced Fc effector functions in protection using *in vivo* models for these livestock diseases to make meaningful conclusions.

## 6 Cellular immune features as correlates of protection

Cytotoxic or killer T cells (CD8+ T cells) and T helper (Th) cells (CD4+ T cells) play a major role in viral immunity (**Figure 1E**). The function of T cells for livestock vaccine studies is commonly measured using IFNγ ELISpot, proliferation assays, and flow cytometry. T-cell response can target both structural and non-structural viral proteins, compared to antibody responses, which are generally more effective against structural proteins.

### 6.1 T cell-dependent antibody response

CD4+ T cells have a key role in generating strong protective humoral immunity through the development of T cell-dependent antibody responses against BTV (211), ASFV (212), and FMDV (213) and should be investigated for CSFV, PRRSV, and LSDV (**Table 1**). Notably, the depletion of CD4+ helper T cells in sheep enhanced clinical signs of BTV primary infection and impaired the development of Nabs, thus highlighting the importance of these cells in the development of protective antibody-mediated immunity (31). The level of T-cell dependency for generating an antibody response is likely antigen dependent (31). However, while BTV-specific CD4+ helper T-cell responses appear to be crucial in the development of a primary antibody response, CD4+ helper T cells were not activated/expanded *in vitro* following stimulation (180), and therefore may play a limited role in the memory response to challenge. Measurement of CD4+ T cells could provide a strong indicator of vaccine failure during vaccine development.

### 6.2 Cytotoxic T lymphocyte response and cell-mediated immunity

Both CD4+ T cells and/or CD8+ T cells can perform antiviral functions through the release of cytotoxic molecules (perforin and granzymes) and cytokines (IFNγ, TNFα, and IL-2), which trigger apoptosis of the infected cell (214). Double-positive CD4+CD8+ T cells are considered a porcine effector memory subset of CD4+ T cells and are strong inducers of cytotoxicity and secretion of IFNγ, and are a major feature in the memory/recall response to vaccination (215). Polyfunctional T-cell responses have been described as important for protective immunity and cross-protection post-vaccination, including proliferation, cytokine secretion, and cytotoxicity for BTV (14, 31), ASFV (87, 92, 93, 182), FMDV (58, 61, 66–68, 216), CSFV (112–114), and PRRSV (42, 47, 217) (**Table 1**). Intriguingly, unlike the other viruses discussed in this review where Nabs—to homologous strains—are likely to be the primary CoP, CMI appears to be the primary CoP for ASFV (**Table 1**) (89, 218, 219). Following vaccination against LSDV, T-cell responses increase; however, the functional role of these cells is yet to be studied (9, 11). The importance of CD8+ T cells in protection from ASFV was highlighted through depletion of CD8+ cells (T cells and NK cells) from pigs immunized against ASFV, resulting in complete loss of protection in these pigs to ASFV challenge, compared to total protection observed in pigs immunized with CD8+ cells (92). Finally, early protection elicited post-vaccination is likely to be cell mediated as observed with the CSFV C-strain LAV, which induced protective immunity in the absence of Nabs, associated with CD4+ T-cell proliferation and IFNγ production (220). While total T-cell responses are commonly assessed, antigen-specific T-cell responses have been rarely characterized against high-priority viral diseases of livestock discussed in this review, and the protective potential of other T-cell subsets remains poorly characterized; for example, γδ (gamma delta) T cells and NK T cells.

### 6.3 T regulatory responses as markers of vaccine failure

It is important to note that some subsets of T cells elicit regulatory functions (221). Known as Tregs, these cells secrete high concentrations of the immunoregulatory IL-10, and excess induction of these responses has been associated with vaccine failure for ASFV (218) and poor clinical outcomes during infection with PRRSV (222, 223). Upregulation of IL-10 following PRRSV vaccination also resulted in reduced induction of an immune response to CSFV vaccination (223). Therefore, secretion of IL-10 by Tregs may act as an indicator of vaccine failure for ASFV and PRRSV. This relationship has not been observed or well characterized following vaccination against FMDV, CSFV, BTV, or LSDV. Further research is needed to elucidate the role of Tregs in protective immunity for disease discussed in this review.

## 7 Discussion

Substantial progress has been made in identifying protective immune features to various vaccine formulations for the high-consequence viral livestock diseases addressed in this review. Nabs are an established CoP for many of these viruses, and a wealth of research has also implicated Type I IFNs and CMI in early vaccine-mediated protection and protection against heterotypic strains of these viruses (i.e., cross-protection) (**Table 1**). However, direct analysis of the correlation to the level of protection for many of these immune features has not been fully established (**Table 1**). Furthermore, the quantitative level of protection for most immune features—except for protective Nab titers against CSFV, FMDV, and PRRSV (**Table 1**)—is not established. The role of an immune feature in protection can be cemented by performing vaccine dose escalation studies in the natural host and correlating level of protection with the immune feature (**Figure 1**). Furthermore, research into the protective role of antibody Fc effector functions, antigen-specific T-cell responses, and cytokine responses to new and existing vaccines is limited. CoP studies in the context of high-priority livestock viral diseases is restricted by reagent availability, lack of protective animal models, and limits to the scale; such experiments can typically be performed due to the practical constraints of large animal studies, compared to human vaccine clinical trials or mice pre-clinical vaccine work. While these limitations are a hurdle for livestock vaccine development, inspiration can be derived from human immune assay development and assessment of vaccine CoPs. Such lessons can be drawn from SARS-CoV-2 research (203, 224–230).

Identification of CoPs has three key applications within a biosecurity system, and these are discussed below. Firstly, identified CoPs can be used to guide the rationale for vaccine development (**Figure 1F**). This can be observed in the abundance of vaccine candidates being developed to generate CMI using DNA or viral vector vaccines against the viruses discussed in this review (122) (**Table 1**). Furthermore, because of high levels of antigenic diversity of strains/genotypes for these high-priority livestock diseases, vaccines are being designed to induce CMI to more conserved proteins to improve cross-protection (87, 231, 232). CoPs in combination with international vaccine standards, which set the minimum requirements for a vaccine, and DIVA capacity should be considered from the beginning of the vaccine development pipeline (233). Secondly, identification of CoPs facilitates selection of an appropriate immune feature to measure for immune surveillance. For example, Nab titers are easy to quantify and protective thresholds can be defined for vaccines that have established Nab titers as a CoP. Nab titers ≥50 following CSFV vaccination in pigs are sufficient to stop virus transmission from wild-type virus challenge, while titers <32 are inadequate to prevent transmission and clinical signs (110). Immune surveillance can be performed to determine the immune status of animals to be transported or exported during an outbreak, identify vaccine failures, and identify at-risk animals that need to be vaccinated (**Figure 1F**). Immune surveillance can be paired with assays to differentiate infected from vaccinated animals to further ensure biosecurity is maintained during trade with regions free of the virus. Finally, a general understanding of the CoP and the level of protection can inform the implementation of a vaccine program (**Figure 1**). For example, the Nab titer of current FMDV vaccines is a reliable indicator of protection against matched strains; however, the correlation between FMDV Nab titers and protection is less predictive of protection to non-matched or heterologous strains (62, 234), thus highlighting the importance of correctly matching the vaccine strain to circulating strains for effective vaccination. This is especially important to consider in regions where multiple virus genotypes or serotypes are circulating (235, 236).

### 7.1 Conclusion

With the emergence, re-emergence, and global movement of high-priority viral diseases of mammalian livestock, understanding CoPs and their applications to biosecurity programs is critical. Identification of CoPs can provide an evidence-based scientific framework to guide next-generation vaccine development and immune surveillance and maintain biosecurity in the livestock industry. Furthermore, a comprehensive understanding of CoPs can facilitate the identification of sub-optimal vaccines or vaccine failures, thus ensuring resilience of livestock populations, promoting global trade, protecting public health, and contributing to sustainable agricultural practices. However, for many important livestock viral diseases, an in-depth understanding of CoPs is lacking, making it difficult to develop improved next-generation vaccines and implement effective vaccination programs for biosecurity. Livestock research needs to take a holistic and comprehensive approach to identifying CoPs using current and novel technologies to drive vaccine development for successful implementation of vaccines in biosecurity strategies.

## Author contributions

SD: Conceptualization, Data curation, Investigation, Project administration, Visualization, Writing – original draft, Writing – review & editing. FJ: Data curation, Investigation, Writing – original draft, Writing – review & editing. QW: Data curation, Investigation, Writing – original draft, Writing – review & editing. MI: Data curation, Investigation, Writing – original draft, Writing – review & editing. AB: Writing – review & editing. DL: Supervision, Writing – review & editing. DW: Supervision, Writing – review & editing. SL: Supervision, Writing – review & editing.
